# Coexistence of a fluid responsive state and venous congestion signals in critically ill patients: a multicenter observational proof-of-concept study

**DOI:** 10.1186/s13054-024-04834-1

**Published:** 2024-02-19

**Authors:** Felipe Muñoz, Pablo Born, Mario Bruna, Rodrigo Ulloa, Cecilia González, Valerie Philp, Roberto Mondaca, Juan Pablo Blanco, Emilio Daniel Valenzuela, Jaime Retamal, Francisco Miralles, Pedro D. Wendel-Garcia, Gustavo A. Ospina-Tascón, Ricardo Castro, Philippe Rola, Jan Bakker, Glenn Hernández, Eduardo Kattan

**Affiliations:** 1 Departamento de Medicina Intensiva, Facultad de Medicina, Pontificia Universidad Católica de Chile, Avenida Diagonal Paraguay 362, Santiago, Chile; 2 Unidad de Cuidados Intensivos, Hospital de Quilpué, Quilpué, Chile; 3 Unidad de Cuidados Intensivos, Hospital Las Higueras, Talcahuano, Chile; 4 Hospital Universitario Puerta del Mar, Cádiz, Spain; 5 Institute of Intensive Care Medicine, University Hospital Zurich, University of Zurich, Zurich, Switzerland; 6 Department of Intensive Care Medicine, Fundación Valle del Lili, Cali, Colombia; 7 Translational Research Laboratory in Critical Care Medicine (TransLab-CCM), Universidad Icesi, Cali, Colombia; 8 Intensive Care Unit, Hopital Santa Cabrini, CIUSSS EMTL, Montreal, Canada; 9 Department of Intensive Care Adults, Erasmus MC University Medical Center, Rotterdam, The Netherlands; 10 Division of Pulmonary, Allergy, and Critical Care Medicine, Columbia University Medical Center, New York, USA

**Keywords:** Fluid resuscitation, Venous congestion, Fluid responsiveness, Critical care, VExUS

## Abstract

**Background:**

Current recommendations support guiding fluid resuscitation through the assessment of fluid responsiveness. Recently, the concept of fluid tolerance and the prevention of venous congestion (VC) have emerged as relevant aspects to be considered to avoid potentially deleterious side effects of fluid resuscitation. However, there is paucity of data on the relationship of fluid responsiveness and VC. This study aims to compare the prevalence of venous congestion in fluid responsive and fluid unresponsive critically ill patients after intensive care (ICU) admission.

**Methods:**

Multicenter, prospective cross-sectional observational study conducted in three medical–surgical ICUs in Chile. Consecutive mechanically ventilated patients that required vasopressors and admitted < 24 h to ICU were included between November 2022 and June 2023. Patients were assessed simultaneously for fluid responsiveness and VC at a single timepoint. Fluid responsiveness status, VC signals such as central venous pressure, estimation of left ventricular filling pressures, lung, and abdominal ultrasound congestion indexes and relevant clinical data were collected.

**Results:**

Ninety patients were included. Median age was 63 [45–71] years old, and median SOFA score was 9 [7–11]. Thirty-eight percent of the patients were fluid responsive (FR+), while 62% were fluid unresponsive (FR−). The most prevalent diagnosis was sepsis (41%) followed by respiratory failure (22%). The prevalence of at least one VC signal was not significantly different between FR+ and FR− groups (53% vs. 57%, *p* = 0.69), as well as the proportion of patients with 2 or 3 VC signals (15% vs. 21%, *p* = 0.4). We found no association between fluid balance, CRT status, or diagnostic group and the presence of VC signals.

**Conclusions:**

Venous congestion signals were prevalent in both fluid responsive and unresponsive critically ill patients. The presence of venous congestion was not associated with fluid balance or diagnostic group. Further studies should assess the clinical relevance of these results and their potential impact on resuscitation and monitoring practices.

**Supplementary Information:**

The online version contains supplementary material available at 10.1186/s13054-024-04834-1.

## Introduction

Current practice guidelines recommend dynamic testing to guide fluid resuscitation [1, 2]. In this context, fluid responsiveness has been defined as a significant increase in cardiac output after a fluid challenge [3]. Approximately, half of the patients admitted to the ICU are fluid responsive (FR+) [4]. Integrating fluid responsiveness assessment into clinical practice is safe [5], avoids ineffective fluid administration to fluid unresponsive patients (FR-) patients [5], and may improve outcomes [6].

However, predicting fluid responsiveness presents certain clinical challenges. First, there are complex technical aspects that may affect the diagnostic accuracy provided by specific tests in different clinical contexts [7]. Second, a common misconception emerges around the objectives of fluid resuscitation that may result in administering fluids until the patient becomes FR- [8]. Finally, there is a general belief that FR+ patients do not develop venous congestion (VC). Recent expert recommendations have stressed the integration of both the concept of fluid responsiveness and prevention of VC, or fluid tolerance, during the early steps of resuscitation (salvage and optimization phases) [9–11]. The final goal is to improve tissue perfusion without increasing the risk of fluid-induced harm, as the presence of VC has been associated with impairment of organ perfusion and organ dysfunction [12–14].

Signals of left- or right-sided VC, such as those obtained from hemodynamic monitoring or bedside ultrasound assessments, could further aid in tailoring this process [15]. These include parameters such as central venous pressure (CVP) measurement [12], estimation of left ventricular filling pressures (such as E/e’ [16]), and abdominal organs venous congestion indexes [17]. In the same line, VC of the lung parenchyma could be estimated by extravascular lung water (EVLW) indexes [18–20] or noninvasively by lung ultrasound [21–23]. Abnormal values of these parameters have been associated with both organ dysfunction and mortality [12, 14, 24–26].

Unfortunately, VC and fluid responsiveness have mainly been studied as separate entities without considering that they may interrelate and share a common ultimate goal, which is a safe and effective fluid resuscitation [27, 28]. There is a paucity of data studying the coexistence of fluid responsiveness and VC in critically ill patients. Understanding this relationship could further aid clinicians in personalizing fluid resuscitation by integrating the benefits and side effects of intravenous fluids into the decision-making process. This could become particularly relevant to the subgroup of patients who are both FR+ and have signals of VC (i.e., fluid intolerant), as in this case, fluid resuscitation could produce more harm than benefit [15].

The main objective of this clinical study was to compare the prevalence of venous congestion signals in critically ill FR+ and FR- critically ill patients after ICU admission. We hypothesized that FR+ patients would present significantly fewer VC signals than FR- patients.

## Materials and methods

We designed a multicenter, prospective, cross-sectional observational study in three medical-surgical ICUs in Chile (Hospital Clínico UC-Christus, Santiago; Hospital de Quilpue, Quilpue, and Hospital Las Higueras, Talcahuano). This study was conducted in accordance with the 1964 Declaration of Helsinki. The ethical review board of each participating site approved this study (CEA-UC No: 220923006; CEC-HGF-SSVQ No: 02/2023; UAIB-HLH-SST No: 3288). The requirement for informed consent was waived owing the observational nature of the study. This report followed the STROBE guidelines for observational studies.

Between November 2022 and June 2023, we included consecutive patients aged > 18 years who required invasive mechanical ventilation and vasopressor support. The exclusion criteria were as follows: a) more than 24 h after ICU admission, b) inadequate echographic window precluding adequate ultrasound assessment, c) mechanical circulatory support, d) pregnancy, e) chronic dialysis [29], f) Child–Pugh C cirrhosis [30], g) prone positioning, and h) any limitation of life support at ICU admission. Eligible patients were assessed at a single time point during the first 24 h after ICU admission, in which fluid responsiveness status and VC signals were measured simultaneously.

Clinical data registered included demographic variables, macrohemodynamic variables, vasoactive drug use, mechanical ventilation parameters, severity scoring such as admission SOFA and APACHE-II, baseline creatinine and tissue perfusion-related variables such as arterial lactate and capillary refill time (CRT), our hierarchical endpoint for resuscitation [31–34]. Central venous pressure (CVP) was measured depending on the presence of a central venous catheter. Clinically relevant outcomes such as ICU length of stay (LOS), hospital LOS, vasopressor infusion, mechanical ventilation, renal replacement therapy duration, development of acute kidney injury (AKI) at day 7, and 28-day mortality were also recorded.

The registered fluid balance (FB) was determined on the basis of the ICU admission pathway. In patients arriving from the emergency room, FB was registered from ER admission to the time point at which the study measurements were performed. In patients who arrived from the operating theater, FB was considered from the induction of anesthesia until the study measurements. Finally, in patients who arrived from the wards, step-down units, or were already in the ICU for other clinical reasons, FB was considered from the initial hemodynamic deterioration until study measurements. Fluid boluses administered in the 6 h after the study measurements and 24 h FB after the study measurements were also registered.

### Fluid responsiveness assessment

Fluid responsiveness was assessed using a pragmatic approach according to the clinical context of the patient, as described in previous studies [35, 36]. The physician could thus decide which was the most suitable test choosing from either pulse pressure variation, stroke volume variation, passive leg raising, or end expiratory occlusion test, considering the presence of arrhythmias, spontaneous ventilation, and the availability of monitoring devices. The cut-off values for each test were defined according to current recommendations [7], and compliance with referred validity criteria was sought actively. Whenever clinical doubts emerged either on the results or applicability of a particular test, a second test was performed to avoid inaccurate measurements.

### Venous congestion assessment

For this study, we included assessments of venous congestion that were noninvasive in nature and readily available at the bedside. Thus, we pragmatically considered two signals of systemic venous congestion, namely CVP and Venous EXcess UltraSound (VExUS) score, and two signals of left-sided venous congestion, the ratio between left ventricular E and lateral e’ Doppler waves (E/e’), and lung ultrasound score [15, 37].

Trained operators performed ultrasound measurements using Mindray M9 (Bio-Medical Electronics Co., Shenzhen, China) and SonoSite Edge II (Fujifilm Sonosite Inc., Bothell, WA, USA) ultrasound machines with concomitant electrocardiogram measurement. Before starting the protocol, all ultrasound operators underwent a training course on the VExUS grading system. Whenever clinical doubts emerged on the ultrasound data interpretation, videos were analyzed by two other independent operators, and consensus was sought. Data obtained from ultrasound measurements were available upon request from the attending physicians.

Transthoracic cardiac ultrasound variables were measured using a phased-array probe. They included the left ventricular outflow tract velocity time integral (VTI-LVOT), shortening fraction acquired from a short parasternal axis, the ratio between the right and left ventricular end-diastolic areas (RV/LV), tricuspid annular plane systolic excursion (TAPSE), E/e’, and inferior vena cava (IVC) maximum diameter.

The VExUS grading system with its independent components (hepatic, portal, and renal veins) was recorded using an abdominal convex probe according to the recommendations by Beaubien-Souligny et al. [17]. An eight-quadrant lung ultrasound score (LUS) was measured. We considered the selected LUS protocol to be both feasible and safe, avoiding potentially hazardous mobilization [38]. Each of the eight anterior quadrants was scanned using a convex probe. Scores were assigned according to the following criteria: 0, A-lines or fewer than two isolated B-lines; 1, multiple well-defined B-lines; 2, multiple coalescent B-lines; and 3, tissue pattern with dynamic air bronchogram [21].

According to previously published cut-off values related to prognostic values, the following were considered as VC criteria: CVP > 12 mmHg [4, 12, 14, 39], LUS > 10 [40, 41], VExUS > 1 [17], and lateral E/e’ > 10 [16, 26]. For study purposes, VC was considered to be present if at least one of these signals was positive. We also compared the prevalence of FR and VC in patients who were categorized as adequately resuscitated or not (according to normal or abnormal CRT status at measurement time).

### Statistical analysis

Based on previous data from general ICU studies reporting fluid responsiveness status and any of the criteria for VC [33, 42], we estimated the prevalence of at least one VC signal in FR- to be 60% and 30% in FR+ patients, respectively. Thus, we calculated the required sample size of at least 84 patients for the trial to provide a statistical power of 80% and an α-error of 0.05.

Data normality was assessed using the Kolmogorov–Smirnov test. Descriptive statistics are presented as median [interquartile range] or percentages. Mann–Whitney U, Student’s t test, chi-square, Fisher’s exact, and z-proportion tests were used when appropriate. Data were analyzed with Prism 10.0 (GraphPad Software, La Joya, CA) statistical package. Two-tailed p-value ≤ 0.05 was considered statistically significant.

As exploratory analyses, we compared the clinical characteristics, fluid administration, and organ support duration of the four groups according to fluid responsiveness and VC status, namely FR+ VC+ , FR+ VC-, FR-VC+ and FR-VC-. We calculated both univariate and multivariate logistic regressions for 7-day AKI, including risk factors with positive univariate associations, such as baseline AKI, SOFA score, and previous fluid balance. Finally, we also assessed the correlation between left- and right-sided signals of venous congestion, namely LUS with E/e’ and VExUS with CVP.

## Results

During the study, 90 critically ill patients were included and followed-up for 28 days. The flow of the study is shown in Additional file 1. Clinical characteristics and outcomes are shown in Table 1. The median patient age was 63 [45–71] years, and the SOFA score at admission was 9 [7–11]. Fluid balance was 1200 [100–2637] ml, norepinephrine dose was 0.11 [0.07–0.25] mcg/kg/min, and 22% required a second vasoactive drug at the time of the study. Forty-one percent of the patients had sepsis as the primary diagnosis on ICU admission, and 22% presented with respiratory failure. Study measurements were performed 7 [1–16] hours after ICU admission.

**Table 1:** Baseline demographic variables, severity scoring, and outcomes of patients included

Variable	Value
**Baseline**	
Age (years)	63 [45–71]
Sex (Female/Male)	49/51%
Weight (kg)	71.5 [64.2–82.5]
APACHE-II score	17 [12–22]
Baseline SOFA score	9 [7–11]
Main admission diagnosis %(*n*)	
- Sepsis	41% (37)
- Respiratory failure	22% (20)
- Surgical	13% (12)
- Neurological	9% (8)
- Hemorrhagic shock	7% (6)
- Decompensated heart failure	2% (2)
- Other	6% (5)
Fluid balance (mL)	1200 [100–2637]
Baseline creatinine (mmoL/L)	1.2 [0.8–1.76]
AKI at admission (KDIGO 1–3) (%)	48% (43/90)
Norepinephrine dose (mcg/kg/min)	0.11 [0.07–0.25]
2nd vasoactive drug use (%)	22% (20/90)
Arterial Lactate (mmol/L)	1.93 [1.3–5.2]
Capillary Refill Time (secs)	3 [2–5]
PaO2/FiO2 ratio	251 [175–335]
PEEP (cmH20)	6 [5–8]
**Outcomes**	
AKI day 7 (KDIGO 1–3) (%)	49% (44/90)
Renal replacement therapy	18% (16/90)
Vasopressor duration (days)	4 [2–7]
MV duration (days)	7 [3–13]
ICU LOS (days)	13 [6–25]
Hospital LOS (days)	25 [14–40]
28-day mortality (%)	21% (19/90)

*PEEP* Positive end expiratory pressure; *AKI* Acute kidney injury; *KDIGO* Kidney disease improving global outcomes; *MV* Mechanical ventilation; *ICU* Intensive care unit; *LOS* Length of stay

The following tests were used to assess fluid responsiveness: pulse pressure variation (65%), stroke volume variation (18%), passive leg-raising maneuver with cardiac output assessment (14%), and end expiratory occlusion test (3%). Thirty-eight percent of the patients were FR+ , while 62% were FR-. 11% of patients required a second test to confirm FR status. At the time of assessment, 18 patients (20%) did not have a central venous catheter in the subclavian or jugular position; therefore, no CVP measurements were available. Patients without CVP measurements were distributed equally between the FR+ (8) and FR- groups (10). All patients had a complete set of cardiac, lung and VexUS ultrasound measurements.

**Fig. 1:**
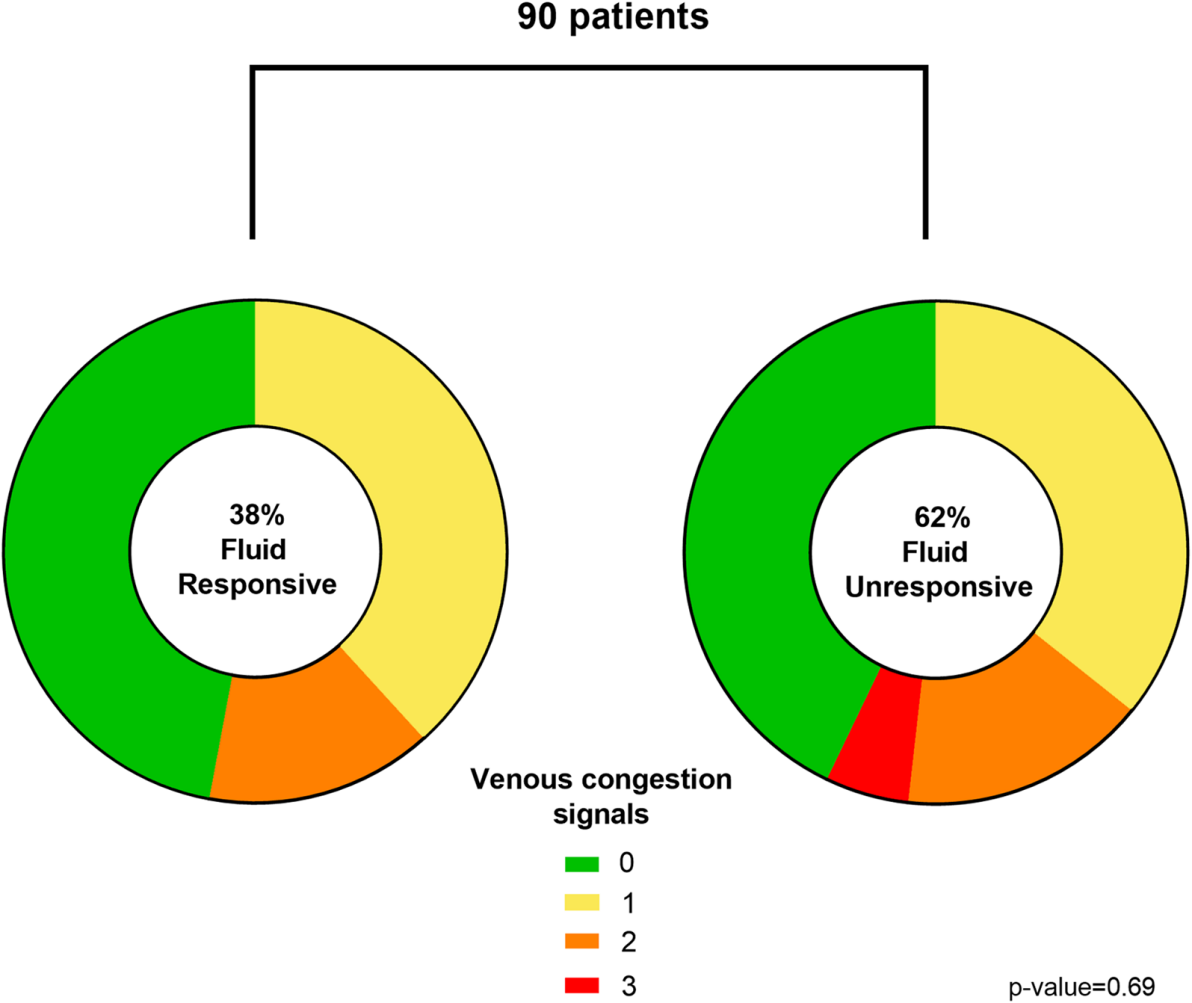
Prevalence of venous congestion signals in fluid responsive and unresponsive patients

The proportion of patients with at least one VC signal was not significantly different between FR+ and FR- groups, as shown in Fig. 1 (53% vs. 57%, *p* = 0.69). The proportion of patients with two or three VC signals was distributed similarly, as well (15% vs. 21%, *p* = 0.4). Additional file 2 shows the prevalence of individual altered VC signals in the FR+ and FR- groups.

**Fig. 2:**
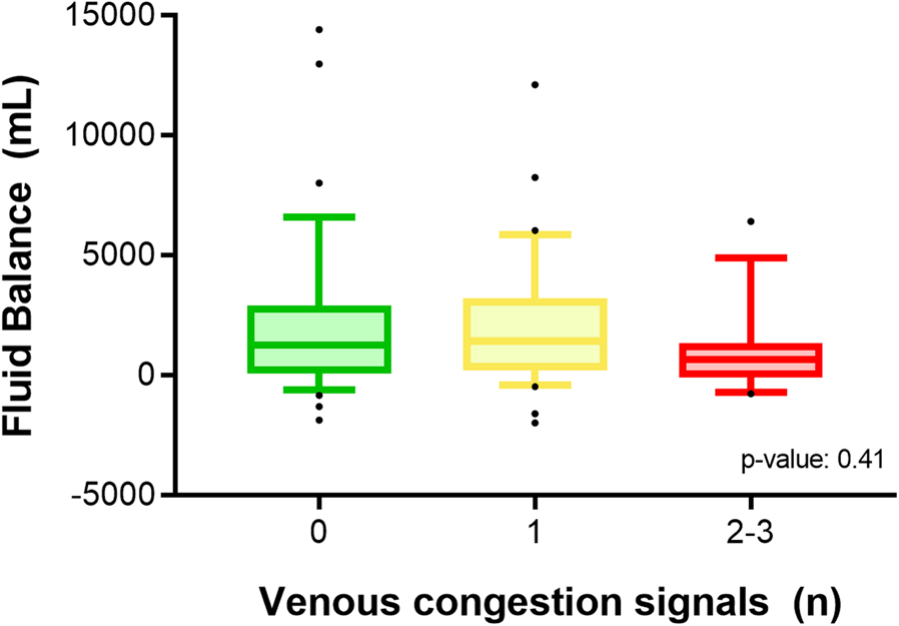
Fluid balance distribution according to the number of venous congestion signals present

Table 2 compares FR+ and FR- patients in terms of key clinical, echocardiographic, and venous congestion variables. Notably, no difference in fluid balance was found among the patients with 0, 1 or 2–3 VC signals (Fig. 2). There were no other statistically significant differences in the key clinical and echocardiographic characteristics between patients with and without venous congestion, as shown in Additional files 3 and 4.

**Table 2:** Key hemodynamic, echocardiographic variables, and venous congestion signals according to fluid responsiveness status

	FR+	FR−	*p*-value
Number	34	56	
Fluid Balance (mL)	1664 [400–3212]	1015 [50–2110]	0.14
Norepinephrine dose (mcg/kg/min)	0.14 [0.09–0.31]	0.1 [0.07–0.22]	0.049
Lactate (mmol/L)	1.8 [1.4–5.8]	2.4 [1.2–4.5]	0.91
CRT (secs)	4 [2–5]	3 [2–4]	0.18
PEEP (cmH2O)	6 [5–8]	7 [5–8]	0.6
Fluid boluses after study measurement (mL)	250 [0–500]	0 [0–0]	0.02
24 h Fluid balance after study measurement (mL)	1100 [− 200 − 2260]	480 [− 950 − 900]	0.046
Baseline creatinine (mg/dL)	1.42 [0.81–2.25]	1.1 [0.8–1.6]	0.24
AKI at admission (KDIGO 1–3) (%)	56% (19)	43% (24)	0.22
**Echocardiographic variables**			
VTI-LVOT	18 [15–20]	21 [16.6–23]	0.03
LV–FAC (%)	60 [50–70]	61 [46–89]	0.68
TAPSE (mm)	19.5 [17.3–23]	20 [18–24]	0.52
RV/LV area > 0.6 (%)	15%	29%	0.2
**Venous congestion signals**			
CVP (mmHg)	9 [4–12]	10 [7–13]	0.45
LUS (*n*)	2 [0–6]	2 [0–8]	0.5
VExUS (*n*)	0 [0–1]	1 [0–1]	0.006
E/e’ ratio	7.1 [5.6–8.7]	6.5 [5.1–8.1]	0.38

*CRT* capillary refill time; *PEEP* positive end expiratory pressure; *VTI-LVOT* velocity time integral of the left ventricular outflow tract; *LV-FAC* left ventricular fractional area change; *TAPSE* tricuspid annular plane systolic excursion; *RV* right ventricle; *LV* left ventricle; *CVP* central venous pressure: *LUS* lung ultrasound score; *VeXUS* Venous excess ultrasound score; *E/e ratio* ratio between early mitral inflow velocity and mitral annular early diastolic velocity

Additional file 5 shows the distribution of FR and VC signals on patients with normal and abnormal CRT at measurement, with similar trends than those of the whole cohort. No significant differences were found on the prevalence of FR+ status (33% vs 44%, *p* = 0.27), any VC signals (53% vs 56%, *p* = 0.83) or 2–3 signals (26% vs 11%, *p* = 0.1) among patients with normal and abnormal CRT.

**Fig. 3:**
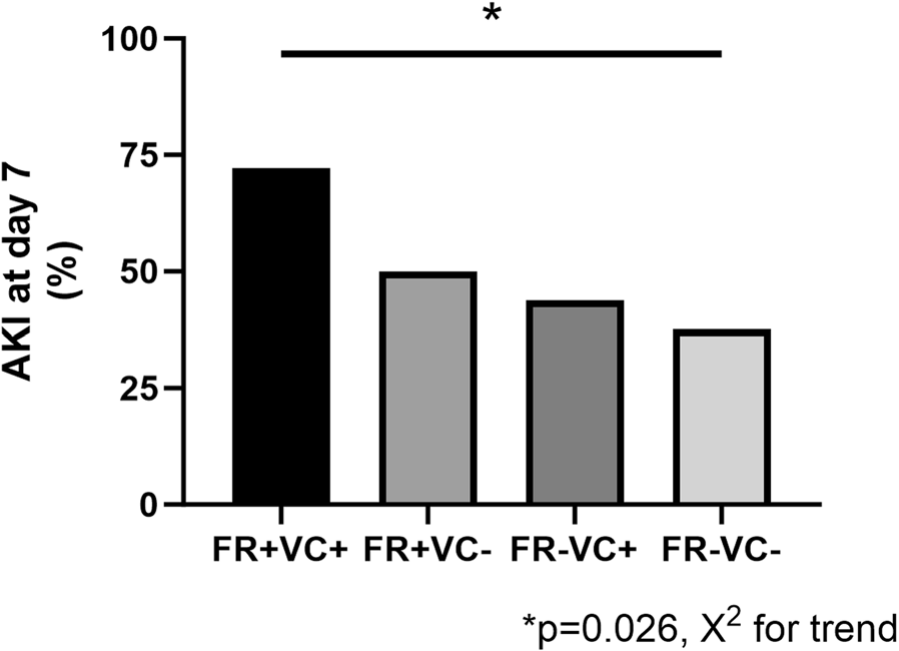
Incidence of acute kidney injury at day 7 according to fluid responsiveness and venous congestion state. FR+ : Fluid Responsive; FR-: fluid unresponsive; VC: venous congestion; AKI: acute kidney injury

An exploratory analysis comparing clinical characteristics, fluid therapy, and organ support of the four groups according to fluid responsiveness and VC status is shown in Additional file 6. The FR+ VC+ group had an OR of AKI on day 7 of 4.33 [1.21–17.4], which was confirmed in the multivariate analysis (Additional file 7). Figure 3 depicts the incidence of AKI on day 7 in the four study groups. Additional file 8 shows that there is a statistically significant difference on CVP readings between patients with normal and abnormal VExUS score, while no difference was found between E/e’ measurements in patients with LUS score higher or lower than 10.

There was no significant difference in the proportion of VC signals between FR+ and FR- patients in the two most prevalent diagnostic groups: sepsis (62% vs. 67%, *p* = 0.75) and respiratory failure (60% vs. 50%, *p* = 0.7) (Additional file 9).

## Discussion

The main results of this study can be summarized as follows: In a cohort of mechanically ventilated patients with acute circulatory dysfunction assessed within 24 h of ICU admission, the prevalence of venous congestion signals was high and independent of fluid responsiveness status. We found no association between fluid balance or diagnosis and the presence of venous congestion signals.

This proof-of-concept study highlights the importance of early assessment of both fluid responsiveness and (fluid) tolerance in acute critically ill patients. Potential fluid intolerance could be identified by early venous congestion signals, such as those used in this study. In fact, different authors have suggested pondering both the beneficial and detrimental effects of fluids in a wide variety of clinical contexts [9, 11, 27, 28]. This becomes particularly relevant in FR+ patients as they are more likely to receive higher amounts of resuscitation fluids because of the common belief that this state precludes fluid-induced harm. In our cohort, unexpectedly, more than half of the FR+ patients could be considered as potentially fluid intolerant as they had at least one VC signal and approximately one-quarter had two or more. Moreover, this subset of patients presented with higher odds of developing AKI at seven days. If confirmed, our results may prompt clinicians to increase their awareness of the potential dangers of fluid administration even in fluid responsive patients.

Even though all patients were assessed during the first 24 h of ICU admission, relying on the temporary framework might provide a rather linear view of the resuscitation process, especially in septic patients or respiratory failure patients, in which the start of the clinical insult is usually unknown and highly variable. Thus, the complementary analysis FR and VC under the optics of the adequacy of resuscitation, according to tissue perfusion signals, could provide an alternative and interesting lecture on the data. Of remark, while the prevalence of FR and VC signals distributed similarly among patients with normal and abnormal CRT (Additional file 5), the clinical interpretation of these results could be divergent. In the former group, the presence of FR- state or the presence of VC signals could trigger clinicians to prompt de-escalation of fluid therapy [2], while on patient still hypoperfused with FR+ and VC signals, deployment of alternative resuscitation strategies could provide a safer resuscitation framework.

Although there is no consensus on how to comprehensively assess systemic or pulmonary congestion, diverse methods have been proposed [37], each with their inherent limitations. In this study, we chose different techniques which had in common that they were readily available and noninvasive, to address the presence of VC. The VExUS score was originally described in postoperative cardiovascular patients and showed strong prognostic significance [17]. However, its use in other scenarios has scant and divergent evidence [43]. Andrei et al. found a prevalence of abnormal VExUS score of 22% in a cohort of general critically ill patients at ICU admission without any correlation with AKI or mortality [44]. In contrast, ultrasound patterns of hepato-splanchnic venous congestion correlated with worse clinical outcomes in acute coronary [45] and septic patients [46]. Even though we found a positive association between altered VExUS and CVP, a known signal of VC and risk factor for adverse outcomes [12, 47], and in line with previous reports [43], future studies should help to determine the best sensitivity, specificity and prognostic values of VexUS components in other high-risk critically ill patients, such as those with septic shock, RV failure or ARDS [48].

Surrogates of ventricular filling pressures, such as lateral E/e´, could aid in identifying left-sided venous congestion. However, this assessment may require advanced technical skills hindering its external applicability [49]. LUS, on the other hand, is a much simpler and accessible technique that correlates well with other signals of VC such as high CVP, high fluid balance and ventricular dysfunction [23, 24]. In our study, even though 20% of patients had altered LUS patterns, median left ventricular filling pressures as assessed by E/e’ values were relatively low, with no association between them (Additional file 8). Thus, the relationship between VC and altered LUS could be impacted by other factors, such as intrinsic respiratory diseases that alter lung permeability or consolidates lung parenchyma [38].

Nevertheless, evidence of lung ultrasounds’ usefulness as a monitoring technique for the resuscitation period could be obtained by the data derived from studies from Caltabeloti et al. In a cohort of patients with septic shock and respiratory failure with relatively low left ventricular filling pressures (similar to our results), they demonstrated the ability of LUS to track rapid changes on lung aeration during resuscitation [50]. LUS increased significantly after 1000 ml of saline were administered in 30 min, a similar pattern as found by Aman et al. who assessed EVLWi kinetics during fluid loading [19]. Thus, dynamic changes could provide valuable insights of potential fluid intolerance.

The high prevalence of VC in FR+ patients at ICU admission could be viewed as a paradox, especially in the current context of moderate positive fluid balances, nonetheless, recent reports from emergency medicine and early ICU admission have shown similar trends [44, 51]. Elucidating why some patients develop early congestion signals, even though they are FR+ , remains challenging. Potential factors include specific disease processes, diastolic or systolic cardiac dysfunction, endothelial and glycocalyx integrity, inflammatory phenomena, and baseline clinical and demographic characteristics [15].This could partly explain why arithmetic fluid balance was not correlated with the presence of VC signals. The fluid tolerance concept [11, 15, 27] provides a novel framework that intends to identify and raise awareness of patients at risk of fluid-induced end–organ damage during the salvage and optimization phases of resuscitation, through available and complementary tools at the bedside [15], which go beyond from relying only on accumulated fluid balance.

There are many limitations that should be mentioned in our study design. First, fluid responsiveness and VC assessments were performed at a single timepoint, without addressing temporal evolution of these parameters. Second, the dichotomization of variables of VC and fluid responsiveness (along with their proposed cut-off values) could be criticized, since they are biological processes with continuous risk distribution; however, this was performed intentionally to answer the research question with a pragmatic approach, and cut-offs were selected based on the association with relevant clinical outcomes, while multiple precautions were taken in both the assessment of FR and VC to increase precision. In the same line, it could be criticized that we did not perform fluid challenges with direct cardiac output assessment to diagnose FR, a highly accurate technique to assess the position of the patient on the Frank Starling curve. Moreover, the observational nature of our study precluded this diagnostic alternative, since the administration of fluids in FR- patients as a diagnostic test could have potential deleterious effects, like inducing or exacerbating fluid-induced harm. In the same line, contemporary research endeavors have used alternative approaches that avoid ineffective fluid administration for diagnostic accuracy studies of fluid responsiveness, diverging as well from these technique [52]. Fourth, other invasive techniques such as continuous cardiac output monitoring, EVLW indexes or quantification of lung aeration through CT scans were not considered as they were not part of routine care in our context.

This study opens the opportunity to better understand the complexities of fluid resuscitation in critically ill patients. Future studies should assess the evolution of fluid responsiveness and VC throughout the resuscitation process, especially in high-risk contexts, such as septic shock, RV failure or ARDS. In the same line, other monitoring variables, either metabolic (i.e., pro-BNP) or derived from advanced cardiovascular monitoring (i.e., EVLWI) could provide relevant information. Finally, integration of these concepts into resuscitation algorithms could provide new tools for the personalization of resuscitation, avoid adverse events, and should be tested in prospective interventional trials.

## Conclusions

Venous congestion signals were prevalent in both fluid responsive and unresponsive critically ill patients. The presence of venous congestion was not associated with fluid balance or diagnostic group. Further studies should assess the clinical relevance of these results and their potential impact on resuscitation and monitoring practices.

## Supplementary Information

**Additional file 1**: Study flow.

**Additional file 2**: Supplemental Fig. 2: Distribution of individual abnormal venous congestion signals according to fluid responsive status. CVP: central venous pressure; VexUS: venous excess ultrasound score; LUS: lung ultrasound score; FR+ : fluid responsive; FR-: fluid unresponsive.

**Additional file 3**: Comparison of clinical variables between patients with and without venous congestion signals.

**Additional file 4**: Key clinical variables according to the number of venous congestion signals.

**Additional file 5**: Distribution of fluid responsiveness and venous congestion signals in patients with normal and abnormal capillary refill time.

**Additional file 6**: Baseline characteristics and organ support of the four subgroups according to FR and VC status.

**Additional file 7**: Univariate and multivariate logistic regressions for 7-day AKI.

**Additional file 8**: Relationship between (A) right-sided venous congestion signals (CVP and VexUS Score) and (B) left-sided venous congestion signals (E/e’ and lung ultrasound score).

**Additional file 9**: Distribution of venous congestion signals in sepsis and respiratory failure patients.

## Data Availability

The datasets are available from the corresponding author on reasonable request.

## Abbreviations

FR+: Fluid responsive
FR−: Fluid unresponsive
ICU: Intensive care unit
VC: Venous congestion
CVP: Central venous pressure
EVLW: Extravascular lung water
EVLWi: Extravascular lung water index
SOFA: Sequential organ failure assessment score
APACHE-II: Acute physiology and chronic health disease classification system II
CRT: Capillary refill time
ScvO2: Central venous oxygen saturation
DpCO2: Venous-to-arterial carbon dioxide difference
LOS: Length of stay
FB: Fluid balance
ER: Emergency room
VTI: Velocity time integral
LVOT: Left ventricular outflow tract
RV: Right ventricle
LV: Left ventricle
TAPSE: Tricuspid annular plane systolic excursion
IVC: Inferior vena cava
VEXUS: Venous excess ultrasound score
LUS: Lung ultrasound score
AKI: Acute kidney injury
SAPS 2: Simplified acute physiology score 2
ARDS: Acute respiratory distress syndrome
Pro-BNP: Pro B-type natriuretic peptide

## References

[1] Evans L, Rhodes A, Alhazzani W, Antonelli M, Coopersmith CM, French C, et al. Surviving sepsis campaign: international guidelines for management of sepsis and septic shock 2021. Intensive Care Med. 2021;47(11):1181–247. 34599691 10.1007/s00134-021-06506-y PMC8486643

[2] Bakker J, Kattan E, Annane D, Castro R, Cecconi M, de Backer D, et al. Current practice and evolving concepts in septic shock resuscitation. Intensive Care Med. 2022;48:148–63. 34910228 10.1007/s00134-021-06595-9

[3] Monnet X, Shi R, Teboul J-L. Prediction of fluid responsiveness. What’s new? Ann Intensive Care. 2022;12:46. 35633423 10.1186/s13613-022-01022-8 PMC9148319

[4] Marik PE, Baram M, Vahid B. Does central venous pressure predict fluid responsiveness? Chest. 2008;134:172–8. 18628220 10.1378/chest.07-2331

[5] Kattan E, Ospina-Tascón GA, Teboul J-L, Castro R, Cecconi M, Ferri G, et al. Systematic assessment of fluid responsiveness during early septic shock resuscitation: secondary analysis of the ANDROMEDA-SHOCK trial. Crit Care. 2020;24. 10.1186/s13054-020-2732-y PMC6979284 31973735

[6] Douglas IS, Alapat PM, Corl KA, Exline MC, Forni LG, Holder AL, et al. Fluid response evaluation in sepsis hypotension and shock: a randomized clinical trial. Chest. 2020;158:1431–45. 32353418 10.1016/j.chest.2020.04.025 PMC9490557

[7] Monnet X, Teboul JL. Assessment of fluid responsiveness: recent advances. Curr Opin Crit Care. 2018;24:190–5. 29634494 10.1097/MCC.0000000000000501

[8] Cecconi M, Hofer C, Teboul JL, Pettila V, Wilkman E, Molnar Z, et al. Fluid challenges in intensive care: the FENICE study: a global inception cohort study. Intensive Care Med. 2015;41:1529–37. 26162676 10.1007/s00134-015-3850-x PMC4550653

[9] De Backer D, Aissaoui N, Cecconi M, Chew MS, Denault A, Hajjar L, et al. How can assessing hemodynamics help to assess volume status? Intensive Care Med. 2022;48(10):1482–94. 35945344 10.1007/s00134-022-06808-9 PMC9363272

[10] Monnet X, Malbrain MLNG, Pinsky MR. The prediction of fluid responsiveness. Intensive Care Med. 2023;49:83–6. 36323911 10.1007/s00134-022-06900-0

[11] Ranjit S, Kissoon N, Argent A, Inwald D, Ventura AMC, Jaborinsky R, et al. Haemodynamic support for paediatric septic shock: a global perspective. Lancet Child Adolesc Health. 2023;7:588–98. 37354910 10.1016/S2352-4642(23)00103-7

[12] Boyd JH, Frcp C, Forbes J, Nakada T, Walley KR, Frcp C, et al. Fluid resuscitation in septic shock: a positive fluid balance and elevated central venous pressure are associated with increased mortality. 2011;39:259–65. 10.1097/CCM.0b013e3181feeb15 20975548

[13] Bagshaw SM, Brophy PD, Cruz D, Ronco C. Fluid balance as a biomarker : impact of fluid overload on outcome in critically ill patients with acute kidney injury. Crit Care. 2008;12:1–3. 10.1186/cc6948 PMC2575565 18671831

[14] Vellinga NAR, Ince C, Boerma EC. Elevated central venous pressure is associated with impairment of microcirculatory blood flow in sepsis: a hypothesis generating post hoc analysis. BMC Anesthesiol. 2013;7:13. 10.1186/1471-2253-13-17 PMC3750825 23919272

[15] Kattan E, Castro R, Miralles-Aguiar F, Hernandez G RP. The emerging concept of Fluid Tolerance : a position paper. J Crit Care. 2022;71. 10.1016/j.jcrc.2022.154070 35660844

[16] Park J-H, Marwick TH. Use and limitations of E/e’ to assess left ventricular filling pressure by echocardiography. J Cardiovasc Ultrasound. 2011;19:169. 22259658 10.4250/jcu.2011.19.4.169 PMC3259539

[17] Beaubien-Souligny W, Rola P, Haycock K, Bouchard J, Lamarche Y, Spiegel R, et al. Quantifying systemic congestion with Point-Of-Care ultrasound: development of the venous excess ultrasound grading system. Ultrasound J. 2020;12(1):16. 32270297 10.1186/s13089-020-00163-w PMC7142196

[18] Jozwiak M, Teboul JL, Monnet X. Extravascular lung water in critical care: recent advances and clinical applications. Ann Intensive Care. 2015;5:1–13. 26546321 10.1186/s13613-015-0081-9 PMC4636545

[19] Aman J, Johan Groeneveld AB, Van Nieuw Amerongen GP. Predictors of pulmonary edema formation during fluid loading in the critically ill with presumed hypovolemia*. Crit Care Med. 2012;40:793–9. 22080639 10.1097/CCM.0b013e318236f2df

[20] Monnet X, Teboul J. Transpulmonary thermodilution: advantages and limits. Crit Care. 2017;21:147. 28625165 10.1186/s13054-017-1739-5 PMC5474867

[21] Mojoli F, Bouhemad B, Mongodi S, Lichtenstein D. Lung ultrasound for critically ill patients. Am J Respir Crit Care Med. 2019;199:701–14. 30372119 10.1164/rccm.201802-0236CI

[22] Mayr U, Lukas M, Habenicht L, Wiessner J, Heilmaier M, Ulrich J, et al. B-lines scores derived from lung ultrasound provide accurate prediction of extravascular lung water index: an observational study in critically ill patients. J Intensive Care Med. 2022;37:21–31. 33148110 10.1177/0885066620967655 PMC8609506

[23] Volpicelli G, Skurzak S, Boero E, Carpinteri G, Tengattini M, Stefanone V, et al. Lung ultrasound predicts well extravascular lung water but is of limited usefulness in the prediction of wedge pressure. Anesthesiology. 2014;121:320–7. 24821071 10.1097/ALN.0000000000000300

[24] Zhao Z, Jiang L, Xi X, Jiang Q, Zhu B, Wang M, et al. Prognostic value of extravascular lung water assessed with lung ultrasound score by chest sonography in patients with acute respiratory distress syndrome. BMC Pulm Med. 2015;15:1–7. 26298866 10.1186/s12890-015-0091-2 PMC4546293

[25] Beaubien-Souligny W, Cavayas Y, Denault A, Lamarche Y. First step toward uncovering perioperative congestive encephalopathy. J Thorac Cardiovasc Surg. 2020;161:149–53. 10.1016/j.jtcvs.2020.02.146 32624312

[26] Abe H, Kosugi S, Ozaki T, Mishima T, Date M, Ueda Y, et al. Prognostic impact of echocardiographic congestion grade in HFpEF with and without atrial fibrillation. JACC Asia. 2022;2:73–84. 10.1016/j.jacasi.2021.10.012 PMC9627800 36340256

[27] Kenny J-ES. Assessing fluid intolerance with Doppler ultrasonography: a physiological framework. Med Sci. 2022;10:12. 10.3390/medsci10010012 PMC8883898 35225945

[28] Argaiz ER, Rola P, Haycock KH, Verbrugge FH. Fluid management in acute kidney injury: from evaluating fluid responsiveness towards assessment of fluid tolerance. Eur Heart J Acute Cardiovasc Care. 2022;11:786–93. 36069621 10.1093/ehjacc/zuac104

[29] Provenzano M, Rivoli L, Garofalo C, Faga T, Pelagi E, Perticone M, et al. Renal resistive index in chronic kidney disease patients: Possible determinants and risk profile. PLoS ONE. 2020;15:1–14. 10.1371/journal.pone.0230020 PMC7112174 32236125

[30] Iranpour P, Lall C, Houshyar R, Helmy M, Yang A, Choi J-I, et al. Altered Doppler flow patterns in cirrhosis patients: an overview. Ultrasonography. 2016;35:3–12. 26169079 10.14366/usg.15020 PMC4701371

[31] Kattan E, Castro R, Vera M, Hernández G. Optimal target in septic shock resuscitation. Ann Transl Med. 2020;8:789–789. 32647714 10.21037/atm-20-1120 PMC7333135

[32] Kattan E, Hernández G, Tascón GO, Valenzuela ED, Bakker J. A lactate-targeted resuscitation strategy may be associated with higher mortality in patients with septic shock and normal capillary refill time : a post hoc analysis of the ANDROMEDA—SHOCK study. Ann Intensive Care. 2020;10:114. 32845407 10.1186/s13613-020-00732-1 PMC7450018

[33] Hernandez G, Ospina-Tascon G, Damiani LP, Estenssoro E, Dubin A, Hurtado J, et al. Effect of a resuscitation strategy targeting peripheral perfusion status vs serum lactate levels on 28-day mortality among patients with septic shock. The ANDROMEDA-SHOCK Randomized Clinical Trial JAMA. 2019;321:654–64. 30772908 10.1001/jama.2019.0071 PMC6439620

[34] Hernandez G, Luengo C, Bruhn A, Kattan E, Friedman G, Ospina-Tascon G a, et al. When to stop septic shock resuscitation: clues from a dynamic perfusion monitoring. Ann Intensive Care. 2014;4:30. 10.1186/s13613-014-0030-z PMC4273696 25593746

[35] Kattan E, Bakker J, Estenssoro E, Ospina-Tascón GA, Biasi Cavalcanti A, De Backer D, Vieillard-Baron A, Teboul JL, Castro RHG. Hemodynamic phenotype-based, capillary refill time-targeted resuscitation in early septic shock : The ANDROMEDA-SHOCK-2 Randomized Clinical Trial study protocol. Rev Bras Ter Intensiva. 2022;34:1–11. 35766659 10.5935/0103-507X.20220004-en PMC9345585

[36] Hernández G, Cavalcanti AB, Ospina-Tascón G, Zampieri FG, Dubin A, Hurtado FJ, et al. Early goal-directed therapy using a physiological holistic view: the ANDROMEDA-SHOCK—a randomized controlled trial. Ann Intensive Care. 2018;8:52. 29687277 10.1186/s13613-018-0398-2 PMC5913056

[37] Melo RH, Santos MHC dos, Ramos FJ da S. Beyond fluid responsiveness: the concept of fluid tolerance and its potential implication in hemodynamic management. Crit Care Science. 2023;35:226–9. 10.5935/2965-2774.20230012-en PMC10406410 37712813

[38] Volpicelli G, Elbarbary M, Blaivas M, Lichtenstein DA, Mathis G, Kirkpatrick AW, et al. International evidence-based recommendations for point-of-care lung ultrasound. Intensive Care Med. 2012;38:577–91. 22392031 10.1007/s00134-012-2513-4

[39] Eskesen TG, Wetterslev M, Perner A. Systematic review including re-analyses of 1148 individual data sets of central venous pressure as a predictor of fluid responsiveness. Intensive Care Med. 2016;42:324–32. 26650057 10.1007/s00134-015-4168-4

[40] Gargani L, Volpicelli G. How i do it: lung ultrasound. Cardiovasc Ultrasound. 2014;12:1–10. 24993976 10.1186/1476-7120-12-25 PMC4098927

[41] Leidi A, Soret G, Mann T, Koegler F, Coen M, Leszek A, et al. Eight versus 28-point lung ultrasonography in moderate acute heart failure: a prospective comparative study. Intern Emerg Med. 2022;17:1375–83. 35181839 10.1007/s11739-022-02943-9 PMC8856869

[42] Castro R, Kattan E, Ferri G, Pairumani R, Valenzuela ED, Alegría L, et al. Effects of capillary refill time-vs. lactate-targeted fluid resuscitation on regional, microcirculatory and hypoxia-related perfusion parameters in septic shock: a randomized controlled trial. Ann Intensive Care. 2020;10:150. 10.1186/s13613-020-00767-4 PMC7606372 33140173

[43] Longino A, Martin K, Leyba K, Siegel G, Gill E, Douglas IS, et al. Correlation between the VExUS score and right atrial pressure: a pilot prospective observational study. Crit Care. 2023;27:1–5. 37237315 10.1186/s13054-023-04471-0 PMC10223840

[44] Andrei S, Bahr PA, Nguyen M, Bouhemad B, Guinot PG. Prevalence of systemic venous congestion assessed by Venous Excess Ultrasound Grading System (VExUS) and association with acute kidney injury in a general ICU cohort: a prospective multicentric study. Crit Care. 2023;27:1–9. 37291662 10.1186/s13054-023-04524-4 PMC10249288

[45] Viana-Rojas JA, Argaiz E, Robles-Ledesma M, Arias-Mendoza A, Nájera-Rojas NA, Alonso-Bringas AP, et al. Venous excess ultrasound score and acute kidney injury in patients with acute coronary syndrome. Eur Heart J Acute Cardiovasc Care. 2023;12:413–9. 37154067 10.1093/ehjacc/zuad048

[46] Fujii K, Nakayama I, Izawa J, Iida N, Seo Y, Yamamoto M, et al. Association between intrarenal venous flow from Doppler ultrasonography and acute kidney injury in patients with sepsis in critical care: a prospective, exploratory observational study. Crit Care. 2023;27:278. 37430356 10.1186/s13054-023-04557-9 PMC10332034

[47] Li D, Wang X, Liu D. Association between elevated central venous pressure and outcomes in critically ill patients. Ann Intensive Care. 2017;7:83. 28795349 10.1186/s13613-017-0306-1 PMC5549673

[48] Prager R, Argaiz E, Pratte M, Rola P, Arntfield R, Beaubien-Souligny W, et al. Doppler identified venous congestion in septic shock: protocol for an international, multi-centre prospective cohort study (Andromeda-VEXUS). BMJ Open. 2023;13: e074843. 10.1136/bmjopen-2023-074843 PMC10373747 37487682

[49] Robba C, Wong A, Poole D, Al Tayar A, Arntfield RT, Chew MS, et al. Basic ultrasound head-to-toe skills for intensivists in the general and neuro intensive care unit population: consensus and expert recommendations of the European Society of Intensive Care Medicine. Intensive Care Med. 2021;47:1347–67. 34787687 10.1007/s00134-021-06486-z PMC8596353

[50] Caltabeloti FP, Monsel A, Arbelot C, Brisson H, Lu Q, Gu WJ, et al. Early fluid loading in acute respiratory distress syndrome with septic shock deteriorates lung aeration without impairing arterial oxygenation: a lung ultrasound observational study. Crit Care. 2014;18. 10.1186/cc13859 PMC4055974 24887155

[51] Prevalska IG, Tucker R, Peter E, Fung CM. Focused cardiac ultrasound findings of fluid tolerance and fluid resuscitation in septic shock. Crit Care Explor. 2023;5: e1015. 38053747 10.1097/CCE.0000000000001015 PMC10695585

[52] Shi R, Moretto F, Prat D, Jacobs F, Teboul JL, Hamzaoui O. Dynamic changes of pulse pressure but not of pulse pressure variation during passive leg raising predict preload responsiveness in critically ill patients with spontaneous breathing activity. J Crit Care. 2022;72. 10.1016/j.jcrc.2022.154141 36116288

