# Supplementary figures and images for: Coexistence of a fluid responsive state and venous congestion signals in critically ill patients: a multicenter observational proof-of-concept study
Source: Crit Care. 2024 Feb 19;28:52. doi: 10.1186/s13054-024-04834-1 (PMC10877871; doi:10.1186/s13054-024-04834-1)

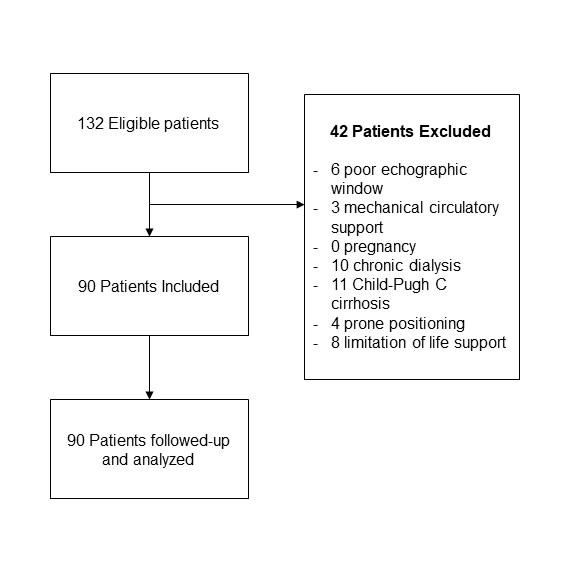

Supplement: Supplementary file 1 — Additional file 1: Study flow. [file 13054_2024_4834_MOESM1_ESM.tif]

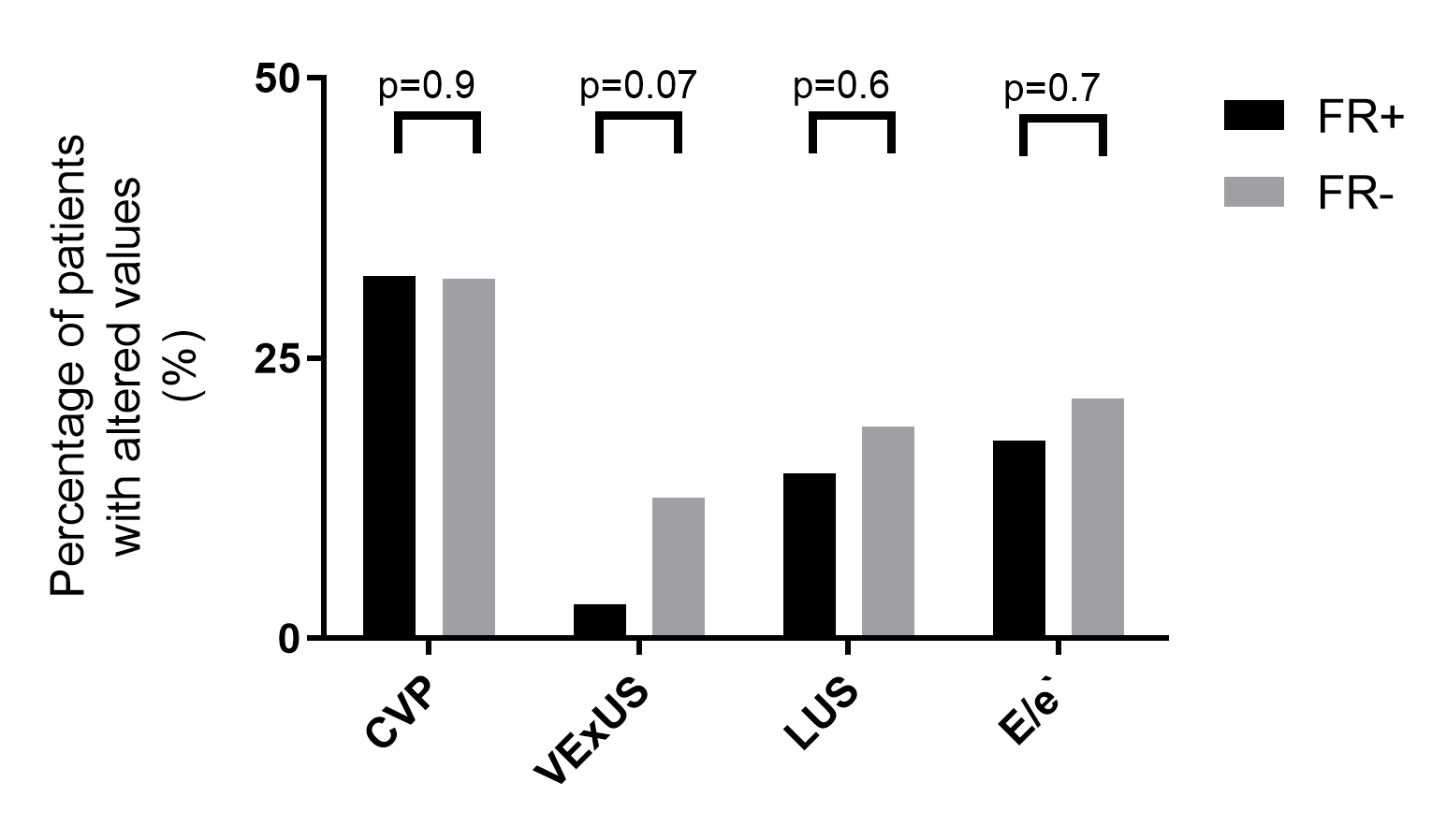

Supplement: Supplementary file 2 — Additional file 2: Supplemental Fig. 2: Distribution of individual abnormal venous congestion signals according to fluid responsive status. CVP: central venous pressure; VexUS: venous excess ultrasound score; LUS: lung ultrasound score; FR+ : fluid responsive; FR-: fluid unresponsive. [file 13054_2024_4834_MOESM2_ESM.tif]

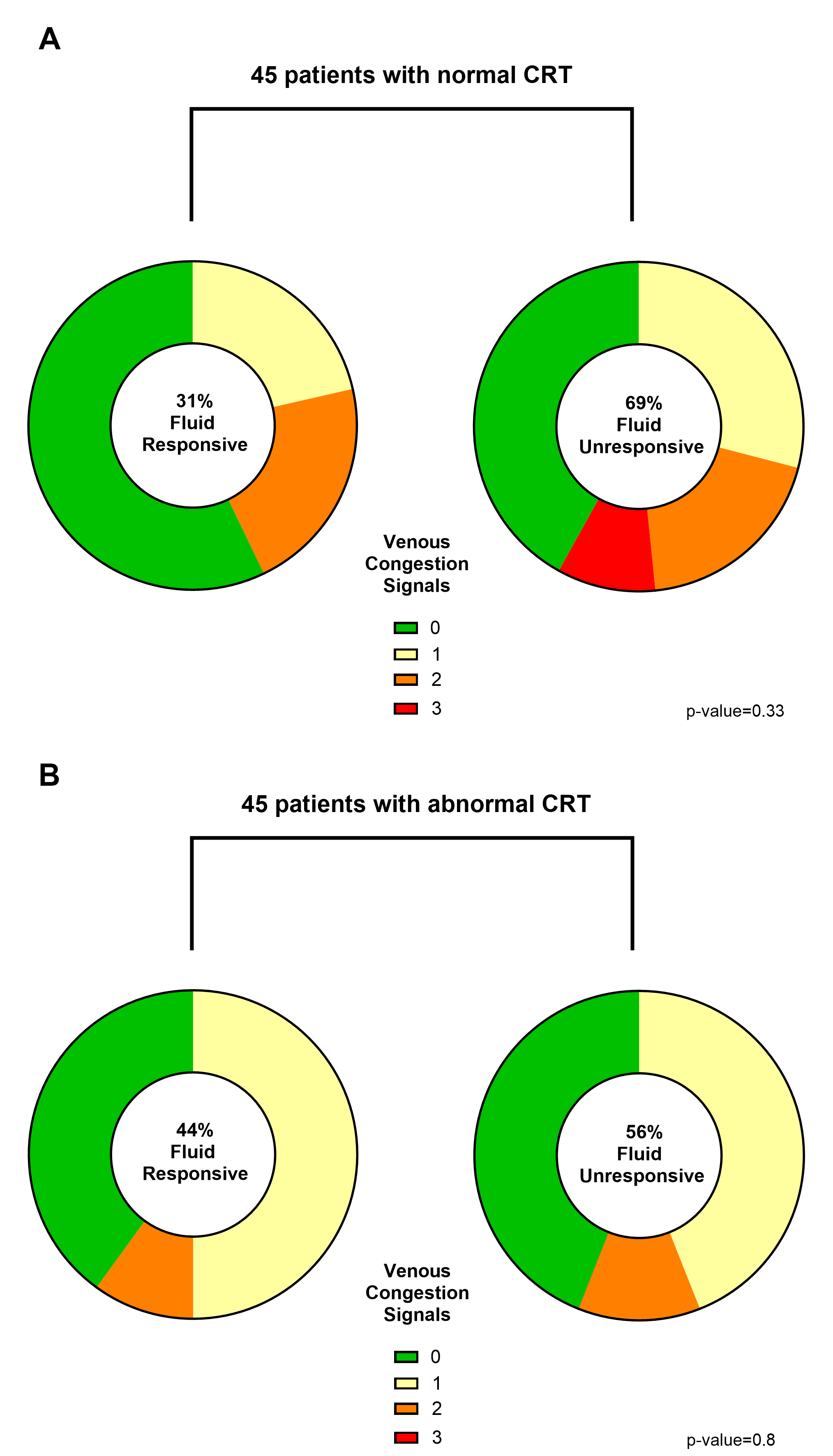

Supplement: Supplementary file 5 — Additional file 5: Distribution of fluid responsiveness and venous congestion signals in patients with normal and abnormal capillary refill time. [file 13054_2024_4834_MOESM5_ESM.tif]

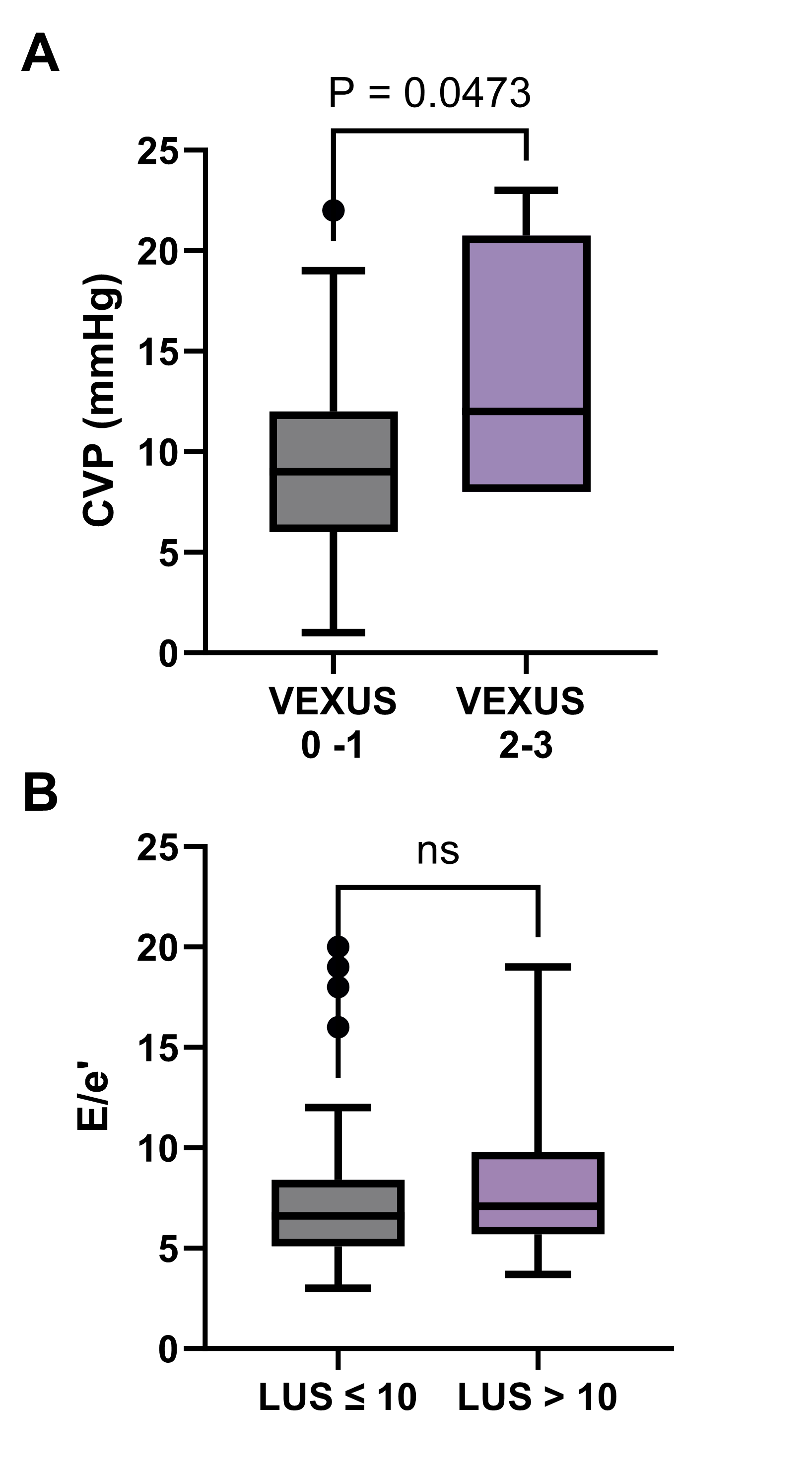

Supplement: Supplementary file 8 — Additional file 8: Relationship between (A) right-sided venous congestion signals (CVP and VexUS Score) and (B) left-sided venous congestion signals (E/e’ and lung ultrasound score). [file 13054_2024_4834_MOESM8_ESM.tif]
