# Supplementary material for: Coexistence of a fluid responsive state and venous congestion signals in critically ill patients: a multicenter observational proof-of-concept study
Source: Crit Care. 2024 Feb 19;28:52. doi: 10.1186/s13054-024-04834-1 (PMC10877871; doi:10.1186/s13054-024-04834-1)
Supplement: Supplementary file 3 — Additional file 3: Comparison of clinical variables between patients with and without venous congestion signals. [file 13054_2024_4834_MOESM3_ESM.docx]

**Additional file 3:** **Comparison of clinical variables between patients with and without venous congestion signals**

|  | No VC signals | VC signals | p |
| --- | --- | --- | --- |
| Age | 59 [43-66] | 67 [49-75] | 0.045 |
| SOFA | 10 [8-12] | 9 [7-11] | 0.2 |
| APACHE | 16.5 [10-21] | 18 [13-23] | 0.41 |
| Norepinephrine dose | 0.1 [0.08-0.22] | 0.12 [0.07-0.27] | 0.65 |
| lactate | 2 [1.3-3.7] | 1.9 [1.2–6.1] | 0.7 |
| CRT | 3 [2-4] | 3 [2-5] | 0.4 |
| C-reactive protein | 10 [3-26] | 17 [7-30] | 0.069 |
| VTI - LVOT | 19 [16-23] | 19 [15-22] | 0.6 |
| LV Shortening Fraction | 60 [50-71] | 60 [45-69] | 0.3 |
| TAPSE | 20 [18-24] | 19 [16-24] | 0.18 |

VC: Venous congestion; SOFA: Sequential organ failure assessment score; APACHE II: Acute physiology and chronic health disease classification system II; CRT: Capillary refill time;

VTI: Velocity time integral; LVOT: Left ventricular outflow tract; LV: Left ventricle; TAPSE: Tricuspid annular plane systolic excursion.
