# Supplementary material for: Coexistence of a fluid responsive state and venous congestion signals in critically ill patients: a multicenter observational proof-of-concept study
Source: Crit Care. 2024 Feb 19;28:52. doi: 10.1186/s13054-024-04834-1 (PMC10877871; doi:10.1186/s13054-024-04834-1)
Supplement: Supplementary file 4 — Additional file 4: Key clinical variables according to the number of venous congestion signals. [file 13054_2024_4834_MOESM4_ESM.docx]

**Additional File 4: Key clinical variables according to the number of venous congestion signals**

|  | 0 | 1 | 2-3 | p |
| --- | --- | --- | --- | --- |
| Age | 59 [43-66] | 63 [48-75] | 69 [48-76] | 0.08 |
| SOFA | 10 [8-12] | 10 [7-11] | 8 [7-9] | 0.16 |
| APACHE | 16.5 [10-21] | 18 [13-23] | 17 [11-24] | 0.7 |
| Norepinephrine dose | 0.1 [0.08-0.22] | 0.13 [0.08-0.32] | 0.1 [0.06-0.19] | 0.13 |
| lactate | 2 [1.3-3.7] | 2.2 [1.2–6.2] | 1.8 [1.2-6.1] | 0.8 |
| CRT | 3 [2-4] | 4 [2-5] | 3 [2-4] | 0.07 |
| C-reactive protein | 10 [3-26] | 16 [10-30] | 22 [4-30] | 0.17 |
| VTI-LVOT | 19 [16-23] | 19[15-24] | 21 [14-22] | 0.8 |
| LV Shortening Fraction | 60 [50-71] | 62 [45-69] | 55 [34-69] | 0.4 |
| TAPSE | 20 [18-24] | 19 [16-23] | 19 [17-25] | 0.6 |

VC: Venous congestion; SOFA: Sequential organ failure assessment score; APACHE II: Acute physiology and chronic health disease classification system II; CRT: Capillary refill time;

VTI: Velocity time integral; LVOT: Left ventricular outflow tract; LV: Left ventricle; TAPSE: Tricuspid annular plane systolic excursion.
