# Supplementary material for: Coexistence of a fluid responsive state and venous congestion signals in critically ill patients: a multicenter observational proof-of-concept study
Source: Crit Care. 2024 Feb 19;28:52. doi: 10.1186/s13054-024-04834-1 (PMC10877871; doi:10.1186/s13054-024-04834-1)
Supplement: Supplementary file 6 — Additional file 6: Baseline characteristics and organ support of the four subgroups according to FR and VC status. [file 13054_2024_4834_MOESM6_ESM.docx]

**Additional File 6: Baseline characteristics and organ support of the four subgroups according to FR and VC status.**

|  | FR+ | | | FR- | | | p-value |
| --- | --- | --- | --- | --- | --- | --- | --- |
|  | VC+ | VC- | p-value | VC+ | VC- | p-value |  |
| N° | 18 | 16 |  | 32 | 24 |  |  |
| Age (years) | 62 [40-68] | 60 [46-67] | 0.9 | 69 [53-76] | 55 [39-66] | 0.01 | 0.09 |
| Diagnosis |  |  |  |  |  |  |  |
| *Sepsis* | 44% (8/18) | 31% (5/16) | 0.4 | 50% 16/32 | 33% (8/24) | 0.2 | 0.8 |
| *Respiratory Failure* | 33% (6/18) | 25% (4/16) | 0.7 | 16% 5/32 | 25% (6/24) | 0.39 | 0.42 |
| *Postsurgical* | 5% (1/18) | 19% (3/16) | 0.3 | 16% 5/32 | 13% (3/24) | 0.9 | 0.62 |
| SOFA score | 10 [8-12] | 9 [8-12] | 0.5 | 8 [7-10] | 10 [7-12] | 0.07 | 0.07 |
| APACHE 2 score | 20 [14-24] | 14 [9-25] | 0.16 | 16 [12-22] | 17 [13-21] | 0.8 | 0.4 |
| Norepinephrine dose (mcg/kg/min) | 0.14 [0.1-0.31] | 0.16 [0.09-0.38] | 0.8 | 0.1 [0.06-0.24] | 0.1 [0.07-0.19] | 0.6 | 0.24 |
| Accumulated Fluid Balance (mL) | 1375 [15-3400] | 1731 [600-3200] | 0.5 | 1110 [150-2050] | 800 [50-2650] | 0.8 | 0.4 |
| 6-h Fluid boluses (mL) | 250 [0-500] | 375 [0-940] | 0.6 | 0 [0-250] | 0 [0-0] | 0.8 | 0.04 |
| 24-h Fluid Balance (mL) | 1100 [-573-1750] | 1000 [-41 – 3211] | 0.5 | -300 [-1150-856] | 550 [-450-960] | 0.09 | 0.18 |
| Creatinine at admission (mmol/L) | 1.48 [1.02-3.7] | 1.0 [0.6-2.1] | 0.06 | 1.23 [0.9-1.6] | 1.1 [0.7-1.6] | 0.38 | 0.11 |
| AKI at admission (KDIGO 1-3) (%) | 67% (12/18) | 43% (7/16) | 0.17 | 47% (15/32) | 37.5% (9/24) | 0.48 | 0.08 |
| CRT (s) | 4 [3-5] | 3 [2-6] | 0.29 | 3 [2-5] | 3[2-4] | 0.9 | 0.3 |
| Lactate (mmol/L) | 1.93 [1.4-6.6] | 1.7 [1.3-3.7] | 0.45 | 2.2 [1.2-6.1] | 2.5 [1.3-3.6] | 0.9 | 0.9 |
| AKI day 7 (KDIGO 1-3) (%) | 72% (13/18) | 50% 8/16 | 0.17 | 44% 14/32 | 37.5% (9/24) | 0.6 | 0.026 |
| Renal replacement therapy (%) | 33% (6/18) | 12.5% 2/16 | 0.23 | 13% 4/32 | 17% (4/24) | 0.7 | 0.22 |
| Vasopressor duration (days) | 5 [3-16] | 3 [2-7] | 0.12 | 6 [2-7] | 5 [2-7] | 0.66 | 0.45 |
| MV duration (days) | 8 [4-17] | 6 [3-17] | 0.7 | 7 [2-11] | 5 [2-13] | 0.7 | 0.7 |

FR: Fluid responsiveness; VC: venous congestion; SOFA: Sequential organ failure assessment score; APACHE II: Acute physiology and chronic health disease classification system II; AKI: acute kidney injury; MV: Mechanical ventilation.
