# Supplementary material for: Coexistence of a fluid responsive state and venous congestion signals in critically ill patients: a multicenter observational proof-of-concept study
Source: Crit Care. 2024 Feb 19;28:52. doi: 10.1186/s13054-024-04834-1 (PMC10877871; doi:10.1186/s13054-024-04834-1)
Supplement: Supplementary file 7 — Additional file 7: Univariate and multivariate logistic regressions for 7-day AKI. [file 13054_2024_4834_MOESM7_ESM.docx]

**Additional File 7: Univariate and multivariate logistic regressions for 7-day AKI**

| Variable | OR [95%CI] | p-value |
| --- | --- | --- |
| **Univariate analysis** |  |  |
| Study group |  |  |
| *FR-VC-* | Reference | - |
| *FR-VC+* | 1.3 [0.44-3.91] | 0.63 |
| *FR+VC-* | 1.67 [0.46-6.2] | 0.43 |
| *FR+VC+* | 4.33 [1.21-17.4] | 0.03 |
| **Multivariate analysis** |  |  |
| Baseline SOFA score | 1.56 [1.13-2.3] | 0.02 |
| Baseline AKI KDIGO 1-3 | 70 [1.08-174] | 0.057 |
| *Baseline AKI*SOFA score* | 0.76 [0.47-1.18] | 0.23 |
| Fluid Balance (L) | 1.1 [0.89-1.38] | 0.4 |
| Study group |  |  |
| *FR-VC-* | Reference | - |
| *FR-VC+* | 3.5 [0.8-16.5] | 0.15 |
| *FR+VC-* | 3.9 [0.7-25] | 0.21 |
| *FR+VC+* | 5.9 [1.14-38] | 0.043 |

FR: fluid responsiveness; VC: venous congestion; OR: odds ratio; AKI acute kidney injury; KDIGO: Kidney Disease Improving Global Outcomes
