# Supplementary material for: Coexistence of a fluid responsive state and venous congestion signals in critically ill patients: a multicenter observational proof-of-concept study
Source: Crit Care. 2024 Feb 19;28:52. doi: 10.1186/s13054-024-04834-1 (PMC10877871; doi:10.1186/s13054-024-04834-1)
Supplement: Supplementary file 9 — Additional file 9: Distribution of venous congestion signals in sepsis and respiratory failure patients. [file 13054_2024_4834_MOESM9_ESM.docx]

**Additional File 9**

**Distribution of venous congestion signals in Sepsis patients**

|  | FR+ | FR- | p-value |
| --- | --- | --- | --- |
| N | 35% (13) | 65% (24) |  |
| VC 0 | 38% (5) | 33% (8) | 0.75 |
| VC 1 | 62% (8) | 50% (12) | 0.49 |
| VC 2 | 0 | 8.5% (2) | 0.9 |
| VC 3 | 0 | 8.5% (2) | 0.9 |
| VC 2-3 | 0 | 17% (4) | 0.27 |
| VC 1-2-3 | 62% (8) | 67% (16) | 0.75 |

FR+: fluid responsive; FR-: fluid unresponsive; VC: venous congestion

**Distribution of venous congestion signals in respiratory failure patients**

|  | FR+ | FR- | p-value |
| --- | --- | --- | --- |
| N | 50% (10) | 50% (10) |  |
| VC 0 | 40% (4) | 50% (5) | 1 |
| VC 1 | 20% (2) | 40% (4) | 0.6 |
| VC 2 | 40% (4) | 10% (1) | 1 |
| VC 3 | 0% (0) | 0% (0) | 1 |
| VC 1-2-3 | 60% (6) | 50% (5) | 1 |

FR+: fluid responsive; FR-: fluid unresponsive; VC: venous congestion
